# Supplementary material for: Are carbon emissions trading and green financial instruments synergistic? -Comprehensive quantitative research based on content analysis
Source: PLoS One. 2024 Mar 7;19(3):e0298601. doi: 10.1371/journal.pone.0298601 (PMC10919601; doi:10.1371/journal.pone.0298601)
Supplement: S1 Data — (ZIP) [file pone.0298601.s001.zip › Data/地级市Z_F工作报告.zip!/┴╔─■/╙¬┐┌╩╨/└┤╫╘╣1⁄2╓┌║┼í╛┬φ┐╦╩2╛▌═°í┐_2014.docx]

各位代表：

现在，我代表市政府向大会报告工作，请予审议，并请市政协委员和列席会议的同志提出意见和建议。

一、2013年工作回顾

过去的一年，在省委、省政府和市委的正确领导下，在市人大、市政协的监督支持下，市政府组织带领全市人民，迎难而上，扎实工作，经济社会保持平稳较快发展。

预计全年地区生产总值完成1500亿元，增长10%；固定资产投资1270亿元，增长17%；社会消费品零售总额389亿元，增长14%；公共财政预算收入184.3亿元，增长8.3%；城镇居民人均可支配收入2.6625万元，实际增长8.3%；农民人均纯收入1.359万元，实际增长9.8%。

（一）工业经济持续健康发展

狠抓以企业为核心的经济运行，实行企业“联系人”制度，为企业提供更好的服务。规模以上工业增加值实现830亿元，增长12%，增速始终保持全省前列。

全市新增规模以上企业102户，达1460户。康辉石化等5户企业主营业务收入超10亿元，总数达24户；新东北电气主营业务收入超50亿元，总数达5户；五矿中板主营业务收入超百亿元，总数达4户，企业实力明显提升。

镁产品及深加工产业集群主营业务收入超800亿元，钢铁及深加工、石化产业集群突破500亿元，纺织服装、食品饮品产业集群超200亿元，汽保装备、包装制品、铝制品及深加工产业集群超百亿。全市17个产业集群实现主营业务收入3030亿元，占全市工业主营业务收入的72%，产业集群建设取得新进展。

全年实施千万元以上重点工业项目720个，其中亿元以上项目307个，拉动工业固定资产投资650亿元，增长36%，工业经济的发展后劲显著增强。

蓝天管件等企业并购罗马尼亚萨瓦公司等9户企业，总数达35户，其中海外企业7户，达23户，成为企业转型升级，实现低成本扩张的重要途径。

全市新增高新技术企业13户，达72户，高新技术产品增加值增长25%。辽宁隆鑫等国家“863”计划项目和国家科技支撑计划项目顺利实施，大石桥获批国家可持续发展实验区，汽保工业园晋升为国家级汽保设备特色产业基地，银珠化纺跻身国家知识产权优势企业行列，卓异集团、盼盼集团和仙人岛能源化工区分别建立了院士工作站，创新驱动发展的能力建设实现新突破。

全市万元工业增加值能耗下降1.7%。

（二）农业和农村经济稳步发展

粮食生产连续十年大丰收，总产量达6.8亿公斤。水稻最高单产和平均亩产继续列全省第1位。

加快“一县一业”建设，盖州市建成水果加工销售龙头企业12家，大石桥市稻米深加工龙头企业达10家，盖州水果、大石桥稻米成为农民增收的可靠途径。

新扩建投资千万元以上农产品加工项目20个，太古糖业等6个投资10亿元以上重大项目全部开工建设，提高了农产品附加值和农业整体效益。

大力发展设施农业、品牌农业，现代农业发展取得长足进步。新建设施小区1万亩、蔬菜基地1.5万亩；新增“三品”认证30个、认定面积10万亩；新增订单农业10万亩，省以上农产品品牌20个，盖州葡萄等4个农产品成功注册国家地理标志保护产品；九寨、杨运苹果种植基地跨入省级出口农产品安全示范区行列；农业机械化综合水平达到75.2%，比上年提高5.4个百分点。农业增效、农民增收的基础更加坚实。

（三）服务业发展呈现强劲态势

进一步下放经济管理权限，激发了城区经济发展活力。服务业增加值占地区生产总值比重达到40%。

服务业集聚区快速发展。大石桥龙街和站前金融集聚区步入省级服务业集聚区行列。温泉、海滨等旅游集聚区持续升温，旅游总收入增长22%，旅游业已成为带动服务业发展的龙头。营口万达广场开工建设，辽宁北方大宗商品交易中心、东北亚镁质材料交易中心建成运营。传统服务业提档升级和现代服务业发展迈出坚实步伐。

“ 夜经济”发展激发了消费活力。开展“营口之夜”主题系列活动，明湖广场、辽河老街、山海广场等“夜经济”特色街区活力凸显，商贸夜间销售额增长20%以上。发展经验在全省推广。

金融业支撑力显著增强。全市新增贷款200亿元，多渠道融资达到300亿元。渤海、阜新银行在我市设立分支机构，机构总数达27家；中融信托在我市设立了庆汇融资租赁公司总部。金融业已成为我市的支柱产业。

加大房屋收储租赁和保障性住房建设力度，促进了房地产业持续平稳发展。

（四）沿海经济带开发开放成效显著

扎实推进港城联动，港城一体化正在形成。港口物流园区加快建设，港前经贸中心和成套装备交易市场一期、现代物流服务中心投入使用，引进物流贸易企业60家，营口港务集团入选中国服务业500强企业。港口金融创新步伐加快，金融大厦等项目开工建设。码头、泊位等基础设施进一步完善，齐齐哈尔等陆港竣工投产。货物吞吐量完成3.3亿吨，增长10%；集装箱运量530万标箱，增长9.3%。港口牵动辐射作用持续增强，吸引了人流，集聚了产业，拉动了城市综合实力快速提升。

重点园区承载能力持续增强。园区基础设施日益完善，完成固定资产投资730亿元，增长21%；新签约落地千万元以上项目228个，新开工建设项目292个，新增投产企业320户。实现规模以上工业增加值274.4亿元，增长13%。

加大出口企业政策扶持力度，地产自营商品出口持续增长。新增自营进出口企业67家，营口耐火材料、保税物流和盖州纺织服装成为首批省级出口基地。全年出口完成44.1亿美元，增长13.5%，出口总量居全省第3位。“走出去”战略深入实施，境外投资企业达24家。

“ 重大项目推进年”活动成果显著。实际利用外资13.3亿美元，增长10%；引进内资589亿元，增长30%。全市开工千万元以上项目1404个。其中，亿元以上项目690个。

投资100亿元的康辉石化、投资35亿元的百威啤酒等一批项目相继投产；

投资151亿元的华威聚酰亚胺、投资60亿元的辽宁海塑新材料产业园、投资50亿元的高威精品铜、投资26亿元的沈鼓透平装备、投资15亿元的无限极健康食品等一批项目开工建设；

投资236亿元的中海石化700万吨DCC、投资50亿元的和润粮油深加工、投资15亿元的正菱工程机械、投资12亿元的棕榈油增塑剂等一批项目签约落地。

这些项目必将成为营口未来发展的支撑力量。

（五）城乡建设和生态环境明显改善

按照“一带三轴”总体布局，编制完成了综合交通、城市CBD等专项规划，规划体系日臻完善。加大老城区改造力度，整治违法建设，市容市貌、环境卫生明显改观。我市被确定为国家智慧城市试点市。

进一步加强基础设施建设，城市功能不断完善。兰旗机场项目进展顺利；大清河、大旱河整治工程有序推进；渤海大街改造等道路工程全部完成；66千伏城东输变电等电网工程投入运行；东部污水处理厂中水回用、市区排水管网改造等重点工程全部完工。这些基础设施的竣工运行，为沿海强市建设奠定了坚实基础。

坚持把生态城市建设作为最普惠的民生工程来抓，着力实施了青山、碧水、蓝天三大工程，生态环境不断改善。森林城市创建步伐加快，植树造林、矿山治理等年度任务全部完成；城镇污水处理厂基本实现稳定达标运行，城市污水处理率达到89%；“气化营口”工程全面实施，扬尘治理取得成效，城市空气优良天数居全省前列。对239个村进行了环境集中综合整治，乡村人居环境明显改观。

（六）民生和社会事业全面进步

10件重点民生工程当年任务全面完成，群众关心关注的一些热点难点问题得到逐步解决。

新增实名制就业6.8万人，城镇登记失业率2.2%，低于省控指标0.8个百分点。新增城镇职工基本养老保险5.8万人，城镇基本医疗保险3.2万人。工伤、失业保险等保障标准进一步提高。新农保和城镇居民养老保险参保率达92%。在全省率先启动新农合大病保险，首创“一卡通”管理模式，参合率达98.8%。农村五保供养标准年提高400元，城乡低保保障标准分别提高12.9%和17.6%，实现应保尽保。东部山区161个贫困村农民人均纯收入增加1200元。就业和社会保障体系建设日臻完善。

学前教育、基础教育、职业教育水平不断提升，教育部批准设立营口理工学院，营口高等教育发展实现了历史性突破。实施文化惠民工程，城乡群众文化活动丰富多彩。文物保护工作不断加强，金牛山遗址被列入全国重点保护大遗址。深化医药卫生体制改革，初步实现城乡居民基本公共卫生服务均等化和农村基层医疗机构国家基本药物制度全覆盖。公共资源交易中心、残疾人康复中心投入运行。出色地完成了马拉松等“十二运”赛事承办工作，展示了营口人的良好风貌和城市的文明形象。

（七）民主法治建设和社会治理进一步加强

自觉接受人大依法监督和政协民主监督，积极听取各民主党派、工商联、无党派人士和各人民团体的意见。人大代表建议和政协委员提案全部办复。深入开展“六五”普法，依法治市、依法行政水平不断提高。进一步改进作风，软环境建设得到改善。

推进“平安营口”建设，强化治安防控体系，打击各类违法犯罪专项行动成效显著，人民群众安全感进一步增强。严格落实安全生产责任制，安全生产形势持续稳定。认真办理群众来信来访，维护了群众合法利益，巩固发展了政治安定、社会稳定的良好局面。

双拥共建、国防教育、民兵预备役和人民防空工作扎实推进；人口和计划生育、统计、新闻出版、国家安全、民族宗教、外事侨务、史志、档案、气象、地震等工作不断加强；慈善、老龄、妇女儿童、残疾人等各项事业取得新成绩。

各位代表：过去的一年，面对错综复杂的经济形势和下行压力，全市上下万众一心，拼搏奋进，在加速率先崛起、全面建成小康社会的征程上留下了坚实足迹。

这些成绩是在多年来持续快速发展的基础上取得的，是省委、省政府高度重视、帮助支持的结果，是市委科学决策、正确领导的结果，是市人大、市政协有效监督、大力支持的结果，是全市干部群众奋发图强、共同努力的结果。在此，我代表市政府，向辛勤工作在各条战线的广大干部群众致以崇高的敬意！向人大代表、政协委员，向各民主党派、工商联、无党派人士和各人民团体，向离退休老领导、老同志，向驻营解放军、武警官兵和中省直单位，向所有关心、支持营口建设和发展的各界人士表示衷心的感谢！

在看到成绩的同时，我们也清醒地认识到，制约营口发展的矛盾还没有很好解决，政府工作还有待进一步加强。受诸多因素影响，部分经济指标没有实现预期目标。经济总量小、债务压力大、财政支撑能力弱的问题还没有解决；产业层次低、丰厚度不高的问题仍很突出；对外开放水平不高，外贸依存度低，与沿海开放城市地位很不相称；节能减排任务十分艰巨，环境保护压力仍然很大；个别部门服务意识不强，工作效率不高，公务员队伍建设尚需加强。对此，我们要在今后的工作中认真加以解决。

二、2014年工作目标与任务

2014年，是贯彻落实十八届三中全会精神、全面深化改革的第一年，也是完成“十二五”规划任务，加速营口率先崛起的关键一年。当前，经济形势依然复杂严峻，下行压力仍然很大，但要看到世界经济开始缓慢复苏，国内经济不断向好，特别是随着改革的全面深化，必将促进生产力的解放和发展，营口正处于新一轮发展的重要机遇期。尤其要看到，经过多年发展，营口的要素资源和区位的比较优势日益明显；“一带三轴”、港城联动的发展格局全面拉开，多点支撑的能力持续增强；经过几年的努力，发展中的一些薄弱环节正在加强，多年积蓄的发展基础和一批重大项目的产能释放效应日趋强劲；几年来，我们攻坚克难，应对挑战，锻炼了一支拼搏进取、干事创业的干部队伍。这是我们发展的坚实基础和有力保障。

推进营口持续健康发展，既要立足当前稳增长、转方式、调结构、惠民生的各项工作，又要着眼长远上水平、提质量、增效益的各项措施，为实现更高质量、更高水平的发展奠定基础。我们坚信，有省委、省政府总揽全局、超前谋划和精心部署，有市委的坚强领导，有全市上下昂扬向上、奋发有为的发展激情，我们一定会把营口发展得更好，让营口人的生活更加幸福。

今年政府工作总的要求是：全面贯彻落实党的十八大和十八届三中全会、中央经济工作会议精神，按照省委、市委十一届七次全会的部署，坚持“稳中求进、稳中求优”的工作基调，改革创新，扩大开放，深入实施“三大战略”，在做大总量中调结构，在优化结构中扩增量，着力提高经济运行质量和效益，着力保障和改善民生，促进经济持续健康发展和社会和谐稳定，在加速率先崛起、全面建成小康社会的征程上迈出新的更大步伐。

全市经济社会发展的主要预期目标是：地区生产总值增长10%，固定资产投资增长19%，公共财政预算收入增长10%，社会消费品零售总额增长14%，城镇居民人均可支配收入增长11%（现价），农民人均纯收入增长13%（现价）。

（一）全面深化各项改革

按照中央、省委、省政府和市委的部署，全面落实好各项改革措施，把中央绘就的改革蓝图变成营口率先崛起的生动实践。

深化经济体制改革。建立和完善土地（林地）经营权流转市场体系，鼓励农民承包经营权在公开市场有序流转。推进科技体制改革，充分发挥企业的创新主体作用和大企业的骨干作用，激发中小企业创新活力。深化国有企业股权多元化改革，妥善推进“壳企业”处置工作。进一步完善现代企业制度。深入推进公用事业领域国企改革，健全考核评价体系，加强服务质量、价格等监管。推进市场准入制度改革，改进工商注册登记制度，实行认缴登记制，鼓励和支持全民创业。积极扶持中小微企业发展，促进个体工商户转企升级。

推进社会事业改革创新。统筹城乡义务教育资源均衡配置，开展中小学校长、教师交流轮岗工作试点；完善文化管理体制，促进由办文化向管文化转变；深化基层医疗卫生机构综合改革，加快公立医院改革；全面实施城镇居民大病保险，推进城乡居民基本医疗保险制度整合。

推进价格改革。完善反映市场供求状况、资源稀缺程度和环境损害成本的价格形成机制。深化水价、电价、气价改革，推进阶梯价格。

推进户籍改革。放开城市户口，促进农业转移人口市民化和外来人口落户。

深化财税体制改革。完善税收征管体制，推进营改增扩面，加强税收征管，确保应收尽收。完善预算管理体制，健全政府非税收入征收管理制度。全面将政府债务收支分类纳入预算管理，把短期应对措施和长期制度建设结合起来，建立健全政府债务风险动态监管机制，加强对政府债务举借、使用和偿还的全过程管控。降低行政成本，压缩“三公”经费，严格执行公务接待标准，严禁铺张浪费；严格控制机构编制，确保财政供养人员只减不增。

加快转变政府职能。调整机关内设机构和职能配置，进一步下放审批权限，减少审批事项，推行网上审批，提高行政效率。改进政府绩效考核制度，继续考核主要经济指标，加大资源消耗、环境损害、生态效益、产能过剩、科技创新、政府性债务、安全生产等指标权重。

（二）扩大对外开放

深入贯彻落实市委《关于进一步扩大对外开放的意见》，坚持外资、内资并重，外贸出口与“走出去”战略并举，全面提升对外开放水平。

围绕世界500强、行业100强抓招商，进一步创新招商体制机制，加强招商队伍建设和软硬环境建设，形成投资洼地；统一招商政策，实行招商选资，突出工业地产和产业集群等主题概念招商，杜绝高耗能、高污染项目；借助上海自贸区和“长三角”地区制造业转移的契机，重点引进具有较强牵动力的科研院所、企业总部和龙头企业。全年实际利用外资增长13%，引进内资增长20%。

围绕建设外贸出口强市抓好对外贸易，以地产自营商品出口为重点，落实好支持出口企业政策，加强出口行业、市场、企业和基地建设。组织重点行业、企业在上海自贸区设立贸易平台，积极开拓国际市场。全年外贸出口增长12%，农产品出口增长20%，外贸依存度达到30%。

围绕加强对外经济技术合作，积极引导优势企业“走出去”，在境外建立生产基地、营销网络和售后服务中心，利用境外资源加快发展，努力实现境外投资新突破。

（三）加大港城联动推进力度

港口是我市最重要的战略资源，也是营口得天独厚的比较优势。坚持以港口为依托，促进港口、产业、城市三位一体协调发展。

加快港口发展。开工建设鲅鱼圈港区疏港公路和仙人岛港区疏港铁路、公路，新建鲅鱼圈港区和仙人岛港区4个泊位。积极开辟外贸航线，大力发展外贸集装箱运输。港口货物吞吐量、集装箱运量均增长10%以上，努力建设外贸大港。

促进港口金融快速发展。制定辽宁（营口）商贸金融创新试验区规划，加快金融创新步伐，完善金融服务功能；推进金融大厦建设，开展金融超市、联保贷款等金融业务，提高港口金融综合服务效率和水平。

改善优化口岸环境。积极申办综合保税区，完成仙人岛口岸对外开放报批。加强“大通关”建设，建立集中报验大厅，改进通关模式，优化通关流程，提高通关效率，确保口岸环境安全、高效、畅通。

大力发展港前产业。加快临港加工业发展，提升鲅鱼圈临港、滨海工业区发展水平。推进港前物流贸易园区、中顺物流园区和大东北农产品物流项目建设。加快成套装备、钢材等专业交易市场建设，促进货值落地，打造区域性商品交易枢纽。抓好东北特产电子商务基地建设，推进沈铁红运多式联运现代物流和电子商务发展，启动实施营港路两侧高端商务区开发建设。

充分发挥港口牵动作用，促进产业生成，完善城市功能，加速人流、物流、资金流集聚，做大城市规模。

（四）全力推进重点园区建设

营口发展的重点在园区、潜力在园区、希望在园区。开展“园区建设年”活动，充分激发每一个园区、每一个要素的发展活力，着力促进产业上规模、园区出形象、项目见效益，把园区建设推向新的更高水平。

围绕项目开工、投产进一步完善基础设施和配套设施建设，促进要素资源向园区集聚。坚持集约节约利用土地，加大占而未建土地清理力度，提高投资强度和产出率。争取产业基地晋升国家级经济技术开发区，高新区创建国家安全产业示范园区。

明晰产业定位，突出产业特色，加速项目集聚、产业集群。着力推动项目开工建设、投产达效，提升园区对全市经济贡献率。重点园区开工千万元以上项目500个，新投产企业490户。中海石化700万吨DCC、中海油1000万吨LNG一期等项目开工建设；辽宁海塑、中船重工等项目投产运行。

加强科技创新能力建设，大力发展新兴产业和高新技术产业，促进结构调整和优化升级。实施重点技术创新项目100项，抓好中小企业共性技术服务平台建设，加快新能源、新材料等新兴产业发展，打造高新技术产业集聚区。高新技术产品增加值占规模以上工业增加值比重达40%。

积极发展海洋经济，培育壮大海洋医药、海工装备制造等海洋特色新兴产业，推广海水工厂化养殖模式，努力打造中国北方海洋产业基地。

深化园区用人、分配和管理制度改革，建立提速发展的体制机制。健全完善园区考核体系，对项目开工投产、债务风险控制、土地集约利用和产出率等指标实行重点考核，促进园区快速发展。

（五）努力提高工业经济发展质量和水平

现有企业是营口发展的基础，要加大支持力度。围绕加快新型工业化，深入实施工业“五项工程”，在规模以上企业全面实行“联系人”制度，强化跟踪服务，为企业发展创造宽松环境。

全年新增规模以上企业100户、主营业务收入10-50亿元企业2户、50-100亿元企业2户，规模以上工业增加值增长12%。

加快推进东旭玻璃基板、北钢管业等260个亿元以上重大工业项目的投产、达产。新开工投资千万元以上项目600个，进一步提升工业经济整体实力。

加强与海内外拟出售的高新技术企业对接，完成并购项目5项，其中海外并购3项，促进企业低成本提质扩张。

积极推进清洁生产、节能降耗，淘汰小镁砂等落后产能，万元工业增加值能耗降低率达到1.5%。

产业集群是企业聚集的旗帜，也是城市品牌。加快产业集群发展，延长产业链条，提高丰厚度，是促进产业优化升级的当务之急。全面推进17个产业集群梯次发展，努力实现重点突破。推进镁产业全面升级，做强精细化工产业，抓好硅藻土、安全智能装备和磁产业发展，打造产业集群新亮点。组建汽保装备、乐器集团公司，加快特色产业集群化发展。全年镁产品及深加工产业主营业务收入突破千亿，钢铁及深加工、精细化工产业均达到600亿元以上，县域重点产业集群主营业务收入均超百亿。

（六）加快发展县域经济和现代农业

调整优化农业结构，加快工业化、城镇化和农业现代化步伐，把农业增效和农民增收落到实处，确保农业基础稳固，农村和谐稳定，农民安居乐业。

既要抓好“米袋子”，又要抓好“菜篮子”，努力实现粮食稳定增产和蔬菜市场价格稳定。大力发展精品农业。突出营口农业特色，加快“三品”认证、认定步伐，新增“三品”认证30个，认定面积10万亩，达到130万亩。全面实施品牌兴农惠民工程，新创省以上品牌10个。大力发展观光农业、设施农业，促进农民稳定增收。完善农机装备结构，加快农业机械化进程。加强农村、海洋气象灾害监测预警体系建设。建立乡镇农产品质量监管站，进一步完善农产品安全保障体系，确保人民群众“舌尖上的安全”。

加快推进“一县一业”，大石桥优质稻米、盖州苹果争取进入省级示范县（市）行列。

抓好农产品加工项目建设，新建千万元以上农产品加工项目20个，太古糖业投产运行。

加大东部振兴战略实施力度。完善政策保障和多元投入机制，大力推进精准扶贫开发和山区移民，扶持发展适宜山区的特色经济，增强山区发展内生动力，确保东部山区农民稳定增收。

（七）大力发展城区经济和现代服务业

加快推进国家现代服务业综合试点市、国家再生资源回收体系建设试点市和现代物流示范市建设，促进服务业提档升级，服务业增加值占地区生产总值比重达到41%。

加快服务业集聚区发展，努力提高集聚度。做强8个省级服务业集聚区，力争主营业务收入全部达到50亿元以上。加快推进营口万达广场、优粤城等服务业高端载体建设，积极发展楼宇经济、总部经济和信息服务、电子商务、文化创意等新兴业态，鼓励工业企业分离发展生产性服务业。

大力发展特色旅游产业。完善旅游产业规划，形成旅游链，使旅游业成为营口经济新的增长点。严格控制温泉资源开发，促进温泉旅游业可持续发展。积极打造温泉、海滨、沟域和乡村旅游特色品牌，开发工业、港口、文化遗址等旅游资源，培育休闲健康产业。整合西炮台、辽河老街、盖州古城等文化旅游资源，抓好影视基地、白沙湾黄金海岸、北海辽东湾海蚀地貌公园建设，打造旅游新热点，使营口成为宜居宜游之地。

促进房地产业持续健康发展。控制开发总量，提升开发档次和品位，加快保障性住房建设，培育房屋租赁市场，保持房地产价格稳定。

繁荣城乡消费市场。抓好兴隆不夜城、辽河老街等夜经济集聚区建设；加强城区农贸市场改造管理；完善农副产品流通体系，促进产供销有效衔接；积极发展家政服务，培育消费热点。

金融业是现代服务业的重要力量，也是经济发展的重要支撑。进一步加快金融业发展，做大经济增长新亮点。抓好多渠道融资，促进银行信贷对经济发展的支持，扩大直接融资比重。积极引进外埠银行业金融机构和投资基金、私募基金战略投资项目，加快企业上市步伐，努力把金融业发展成为现代服务业的强力支撑。

（八）加强城市和生态环境建设

加强规划引领和约束，完善规划管理制度，严格规划执法检查，维护规划的严肃性、权威性。

加大基础设施建设力度，完善城市功能，使人民群众的生活条件有一个大的改善。全力推进兰旗机场建设。做好城市轨道交通前期规划，完成“外环路”外迁改造工程和高铁营口东站东出口建设。实施大辽河主城区段防洪治理工程，完成大旱河、大清河等河流整治改造任务，水毁工程年内全部完工。实施13项输变电工程，推进热电工程建设。扩大集中供热面积，集中供热率达到92.6%。加快城市“北转”步伐，改善人居环境。

加快“智慧营口”建设，提升城市信息化、精细化管理水平。完善“大城管”格局，严格城市管理执法，积极开展市容市貌专项治理，加大违法建设、占道经营整治力度。完善交通设施，开展道路交通安全整治行动，规范交通行为，维护交通秩序。加强市政设施维护，引导市民关爱城市环境，积极创建全国卫生城市，共同打造美丽家园。

突出文化传承和地域特色，稳步推进新型城镇化。抓好小城镇试点，集约节约城镇建设用地，争取进入国家城镇化建设试点市。抓好产业、就业支撑，使城镇化与工业化、信息化和农业现代化统筹推进；抓好人口集聚，有序推进农业转移人口市民化，促进城镇化与新农村建设协调发展。

加大“三项工程”实施力度，努力建设山青、水秀、天蓝的生态型城市。推进城乡造林绿化，加强破损山体和闭坑矿、生产矿治理，把营口建设成为国家级森林城市；加强饮用水源地保护，封闭地下水取水工程全部结束；加快西部污水处理厂、开发区污水处理厂提标改造及大石桥污水处理厂二期等工程建设，使营口流域水质有一个大的提升；加强雾霾监测预警，推进“气化营口”建设，加快公交车、出租车“油改气”进程，提升空气质量。

巩固农村环境集中综合整治成果，启动227个村的环境整治工程。健全完善农村环境管理长效机制，高标准建设一批环境整洁、管理完善、文明有序的美丽乡村。

（九）大力推进民生改善和社会事业发展

启动实施主城区集中供热、老城区环境提升等12件重点民生工程，推进平价商店等民生项目。加大动迁居民安置力度，主城区没有及时回迁的动迁户力争全部安置完毕。

积极扩大就业，全年新增实名制就业7万人，城镇登记失业率控制在2.8%以内。抓好失地失海人员、残疾人等重点和特殊群体就业；加大复员退伍军人安置力度，兜底安置困难家庭未就业高校毕业大学生，缓解我市各类人才短缺问题。适时提高各项养老金和保险待遇标准，确保及时足额发放。加快构建养老服务体系，居家养老服务中心力争年内投入运营。

大力发展社会各项事业。推进学前教育乡村一体化品牌连锁发展，加快市属学校标准化建设，巩固义务教育强县（区）成果，推进中等职业教育园区建设。营口理工学院努力创建高水平、有特色的本科院校。加快发展文化事业，推进市图书馆、档案馆建设，完善公共文化服务体系，活跃城乡文化市场。大力培育印刷包装、影视创作、文化创意等文化产业新亮点，不断提升文化软实力。加快市第二人民医院、第一专科医院、市公共卫生服务中心建设。提高新农合保障能力和服务水平，新农合参合率稳定在98%以上，推进城乡居民公共卫生服务均等化。实施农民健身工程，提高全民健康水平，努力创建全国文明城市。

加强和创新社会治理。深入开展“六五”普法，推进依法治市。实施网上公安工程，健全社会治安综合防控体系，建设“平安营口”。严格落实安全生产责任制，促进安全生产形势持续稳定。严格执行重大决策社会稳定风险评估机制，完善大信访工作格局，维护社会和谐稳定。建立统一高效的食品药品监管体制，保障人民群众饮食用药安全。

加强国防教育、民兵预备役建设，积极开展双拥共建活动。做好经济普查和统计工作，抓好物价监管、人民防空、人口计生、新闻出版、国家安全、民族宗教、外事侨务、史志、档案、气象、地震等工作。积极发展慈善、老龄、妇女儿童、残疾人、未成年人等各项事业。

（十）加强政府自身建设

扎实开展党的群众路线教育实践活动，转变作风，提高效率，努力建设人民满意政府。

自觉接受人大法律监督、政协民主监督和社会各界监督，认真执行人大及其常委会各项决议决定，认真办理人大代表的建议、意见和政协提案，提高满意度和落实率。

大兴学习之风，加强能力建设。坚持以党的十八大、十八届三中全会和习总书记系列重要讲话精神武装头脑，加强理论、政策和业务知识学习，不断提高科学发展能力、开拓创新能力、维护社会稳定能力和处理各种复杂矛盾的能力。

自觉维护法律尊严，坚持依法行政。建立健全责任追究和绩效考评制度，使政府各项工作规范化、制度化。加强行政执法监督，落实行政执法责任制，把依法行政贯穿于政府决策、执行、监督全过程。

开展“作风建设年”活动，转变工作作风。完善反对“四风”的长效机制，做到“讲诚信、懂规矩、守纪律”；始终保持奋发有为的发展激情，勇为人先，敢于担当，大胆实践；弘扬脚踏实地、严谨求实作风，对每项工作一件一件抓落实，一个项目一个项目抓成效，提高执行力、公信力，以实实在在的业绩，让人民满意。

坚持从严治政，廉洁从政。严格执行党风廉政建设责任制，加强理想信念教育，强化监督管理，严惩腐败行为。深入落实中央“八项规定”和省委、市委的“十项规定”，率先垂范，廉洁自律，建设廉洁政府。

各位代表：我们面临的经济形势依然严峻，工作任务艰巨而繁重。我们有基础、有能力、有自信，完成好确定的各项目标任务，推动全市经济社会持续健康发展。让我们在省委、省政府和市委的正确领导下，戮力同心，奋发图强，共同创造营口人民更加幸福美好的未来！
